# Characterization of an Acute Rodent Osteomyelitis Infectious Model Using a Tibial Intramedullary Implant Inoculation

**DOI:** 10.3389/fbioe.2020.567647

**Published:** 2020-10-09

**Authors:** Michel Assad, Anne Marie Downey, Caroline Cluzel, Yannick Trudel, Nancy Doyle, Simon Authier

**Affiliations:** ^1^Charles River Laboratories, Boisbriand, QC, Canada; ^2^Charles River Laboratories, Laval, QC, Canada

**Keywords:** osteomyelitis model, intramedullary, orthopedic implant, biomaterials, periprosthetic, antimicrobial testing, infection, bone histopathology

## Abstract

Chronic osteomyelitis in presence of orthopedic implants is a condition observed in the field of biomaterials as it impairs early bone-implant contact, fixation and integration. In this study, a surgical intramedullary tibial insertion was performed using a titanium wire previously inoculated with *Staphylococcus aureus* in order to develop an osteomyelitis model in a clinically relevant long bone and in absence of any prophylactic treatment. As such, twenty-two male Sprague-Dawley rats received a sterile or inoculated intramedullary biomaterial with either 2 × 10^6^ or 1 × 10^7^ *S. aureus* colony forming units. Bacterial burden, inflammation, morphological changes, as well as newly formed bone tissues were evaluated for histopathology following a period of either eight or fifteen days of implantation. The implant inoculated in presence of the highest bacterial load was effective to produce significant periprosthetic infection observations in addition to hard and soft tissue inflammation consistent with the development of osteomyelitis. In contrast, neither the sterile nor the low-dose implant inoculation showed inflammation and clinical infection signs, but rather produced an expected bone remodeling and appropriate healing associated with biomaterial implantation. Complete health assessment is presented with histopathological periprosthetic results.

## Introduction

Chronic orthopedic periprosthetic joint infection is a recognized complication in biomaterials science since it has an impact on early implant integration, fixation and osseointegration. Although there is a relatively low rate of primary infection (1.5–2.5%) associated with total joint arthroplasty (Lentino, 2003; Puckett et al., 2010; Cram et al., 2012), recurrence of infection is relatively high (15–40%) with traditional invasive orthopedic implants (Lentino, 2003; Alt et al., 2014; Varrone et al., 2014). Gram-positive *Staphylococcus aureus* bacteria (*S. aureus*) represent important clinical pathogens and are considered one of the most common etiologic agent of implant-associated chronic osteomyelitis following invasive orthopedic surgeries including total hip and knee replacements, and spinal procedures (Lentino, 2003). Non-curable infections can in turn lead to non-union, implant loosening and ultimately to the early implant failure with a possible requirement for revision surgery (Lerouge et al., 1996; Lentino, 2003; Lichstein et al., 2014). As a result, it is estimated that 8–15% of total joint arthroplasty failures are directly related to an infection (Lentino, 2003; Kurtz et al., 2008; Puckett et al., 2010). With changes to patient demographics, the incidence is expected to augment as elective surgeries are also predicted to increase with prosthetic device wear over time. This will necessarily represent a significant economic burden on our healthcare system and society with consequent economic implications (Kurtz et al., 2008, 2012; Cram et al., 2012; Hernández-Vaquero et al., 2013).

It also seems that chronic osteomyelitis may result from the emergence of methicillin-resistant *S. aureus* (MRSA) that is responsible for typically 50% of the cases of surgical and nosocomial infections (Kurtz et al., 2008; Varrone et al., 2014; Nishitani et al., 2015). Indeed, an early emergence of *S. aureus* and a subsequent increase in antimicrobial resistance is observed when traditional prophylactic therapy fails to prevent infections (Vergidis et al., 2011). This is due to bacterial capacity to induce the formation of a biofilm that occurs within bone tissue and its apposed implant even in presence of standard antibiotic therapy (Brady et al., 2008; Puckett et al., 2010; Arciola et al., 2012; Odekerken et al., 2013; Nishitani et al., 2015). This biofilm may represent an ideal condition for bacterial growth while being shielded from the immunological response and therapy according to Nishitani et al. (2015). Surface-adherent bacteria with cell wall molecules, called adhesins, detect mammalian structures, attach to the implant surface, then proliferate and secrete various matrix proteins (Patti et al., 1994; Nishitani et al., 2015). Bacterial isolates expanding in biofilms present poor antimicrobial sensitivity compared to other forms (Vergidis et al., 2011). Biofilm remodeling may rely on a gene regulator-dependent bacterial dissemination mechanism that maintains chronic infections, which in turn inhibits adequate implant osseointegration with periprosthetic tissue (Puckett et al., 2010; Nishitani et al., 2015). The mode of action by which hard tissue infection develops has been further explored. de Mesy Bentley et al. (2017) have noticed that residual bacteria are present in infected cortical bone and show a mechanism of motility by which *S. aureus* can identify bone canaliculi, deform from cocci into rod-shaped cells and convey through these channels.

In order to better understand the bacterial microenvironment and possible treatment, various animal osteomyelitis models have been developed over the years. For example, Nishitani et al. (2015) have investigated some of the proliferation characteristics of *S. aureus* biofilm growth to better understand host and pathogens that regulate this process by exploring the *in vivo* generation of biofilm using SEM and bioluminescence. Compromised animal implantation models for this disease remain an effective method to assess the safety and performance of newly developed prophylactic therapies including coated biomaterials (Alt et al., 2014). In the present experimental study, an osteomyelitis infectious rat model was evaluated using titanium implantation. As such, a surgical intramedullary tibial insertion was performed using inoculated pins with a *Staphylococcus aureus* bacterial load to produce an acute infection result in absence of prophylactic antibiotics administration following de Mesy Bentley et al. (2017) and Andriole et al. (1974) procedures. One specific objective was to confirm the inoculation dose of *S. aureus* in a clinically relevant long-bone region of interest. Complete health assessment, in addition to qualitative histopathological and radiological results are presented on the local and systemic effects of inoculated implants for which absolute incidence is reported. Bacterial burden, inflammation, morphological changes, and newly formed bone tissues were also evaluated following the course of a 15-day implantation study.

## Materials and Methods

### Biomaterials and Study Design

Biomaterials for disease induction were represented by intramedullary pins [Ti-6Al-4V titanium alloy TI Kirschner wires (K-wires); Ø1.0 mm × 150 mm; DePuy Synthes, Monument, CO, United States)], which are traditionally used in orthopedics to help reduce and stabilize fractures, osteotomies and fusions. In this study, wires were cut to 10-mm lengths and sterilized with autoclave (automatic sterilizer, model 3870EA; Tuttnauer, Breda, Netherlands) at 134°C for 3 min. Either sterile (Group 1), low-load (Group 2) or high-load inoculated titanium K-wire implants (Group 3; Table 1) were then surgically inserted in the intramedullary cavity as described below.

**TABLE 1:** Study design.

Treatment Group	*S. aureus* Dose (CFU)	Number of Animals	Necropsy (Day 8*)	Necropsy (Day 15)
Control Sterile Implant (Group 1)	0	N = 10	N = 5	N = 5
Low-Dose Inoculated Implant (Group 2)	2 × 10^6^	N = 5	N = 3	N = 2
High-Dose Inoculated Implant (Group 3)	1 × 10^7^	N = 7	N = 4*	N = 3

*Due to a condition resulting from a postsurgical complication, one animal was necropsied on day 8 instead of day 15.

### *Staphylococcus aureus* Strain Inoculum and Implant Preparation

A *Staphylococcus aureus* cell strain culture (ATCC^®^ 6538^TM^; American Type Culture Collection; ATCC, Manassas, VA, United States) was used as an inoculum prior to surgical implantation. This bacterial species is well characterized because it induces infection *in vivo* (Odekerken et al., 2013; Nishitani et al., 2015). The organisms were grown in tryptic soy broth (TSB; Alpharmco Laboratory, Montréal, QC, Canada) at 37°C with intensive shaking at approximately 150 rpm for 12–18 h and then diluted with sterile saline in order to achieve a density of 1 × 10^7^ colony forming units (CFUs)/mL. The bacterial inoculum was selected based on the published article by de Mesy Bentley et al. (2017). Following sterilization, some of the pins were exposed to 1mL of culture of *Staphylococcus aureus* with either 2 × 10^6^ (lower inoculation load) or 1 × 10^7^ CFUs (higher inoculation load) for 30 min, prior to intramedullary insertion into the tibiae to create a clinically representative model of periprosthetic infection. Other K-wires were kept sterile as unexposed control implants.

### Animal Model, Diet and Environment

The rat has been selected for this study as it is a universally used rodent standard species for regulatory toxicity testing to evaluate the safety and efficacy of various classes of biomaterials and for which, there is a large historical database. The intramedullary implantation is one of the potential routes of human exposure to *Staphylococcus aureus* infection caused by a foreign body in orthopedic arthroplastic surgery. In this study, 22 male Sprague-Dawley rats [Crl:CD(SD)IGS; *Rattus norvegicus*; 458–500 g; 13 weeks of age; Charles River Canada Inc., Saint-Constant, QC, Canada] were first subject to a detailed clinical examination by an attending veterinarian to ensure that animals were healthy and suitable for use on the study.

A period of 7 days of acclimation was then allowed before surgery in order to accustom the animals to the laboratory environment. During the acclimation period, animals were randomly assigned to either treatment or control groups (Table 1). Immediately following surgical procedures, animals were returned to their respective cages, which were clearly labeled with a cage card indicating the study and animal numbers. Municipal tap water, which was exposed to ultraviolet light and purified by reverse osmosis, was provided to the animals *ad libitum* via an automatic watering system. Similarly, a standard certified commercial diet (Teklad Certified Global Rodent Diet #2018C; Envigo RMS Division, Indianapolis, IN, United States) was also provided to the animals *ad libitum*.

The animal room environment was set to maintain a temperature of 21 ± 3°C, a relative humidity of 50 ± 20%, a light dark cycle of 12 h light/12 h dark, except during designated procedures, and 10–15 air changes per hour. All animals were observed at least twice daily and detailed clinical exams were performed at least weekly throughout the study period. Technical staff and veterinarians were available 24 h per day for treatment of any untoward clinical signs.

### Ethical Considerations, Animal Welfare and Care

Protocols, procedures and husbandry including strict euthanasia criteria involving the care and use of animals in this study were reviewed and approved by the institutional animal care and use committee (IACUC) of a Charles River Laboratories preclinical testing facility accredited by the Association for Assessment and Accreditation of Laboratory Animal Care (AAALAC). All experimental procedures were also performed in accordance to the Canadian Council on Animal Care (CCAC) guidelines for use of experimental animals especially helpful for infection studies, which prescribe to replace animals when they are not necessary, reduce the number of animals enrolled on study to its minimal, as well as refine and adapt husbandry for minimal pain and distress possible. Also importantly, biomaterial implantation for osteomyelitis creation, animal and tissue handling including necropsy followed precautions of Biosafety Level II standards.

### Animal Preparation

Analgesia (buprenorphine: 0.3 mg/mL, 0.4 mL/animal; Champion Alstoe Animal Health Inc., Sheriff Hutton, York, United Kingdom) was administered by subcutaneous injection at least 30 min prior to surgery and twice daily for five postoperative days for pain management. An ophthalmic ointment (Systane^®^; Alcon, a Novartis Company, Dorval, QC, Canada) was applied to both eyes to prevent drying of the cornea before and following surgery. Anesthesia was induced with isoflurane induction (3%; 3L/min O_2_; Fresenius Kabi AG, Bad Homburg, Germany) and isoflurane maintenance (1%; 0.5L/min O_2_; Fresenius Kabi AG, Bad Homburg, Germany). The right hindlimb was then shaved using electric clippers and aseptically prepared for tibial exposure using a 4% chlorhexidine solution (Hibitane; Partnar Animal Health, Ilderton, ON, Canada). The surgical site was cleaned topically with a 1% iodine solution (Teva Canada Ltd., Toronto, ON, Canada) as needed to the exposed skin. Fluid therapy (sterile NaCl 0.9%, USP, 2.0 mL subcutaneous; Baxter Healthcare, Deerfield, IL, United States) was provided and an Elizabethan collar (Ludomed Equipment Inc., Saint-Zotique, QC, Canada) was then used as needed to prevent the animals from accessing the implantation site.

### Surgical Approach

The right hindlimb was flexed to a position to which the medial condyle of the femur could be palpated under the skin. A parapatellar incision was performed and the subcutaneous tissues were dissected to reveal the femoral condyle and identify the patellar ligament. The superior cortex of the tibial plateau was then perforated, just medial to the patellar ligament. As predrill, a sterile Kirschner wire was first driven distally in the intramedullary canal, approximately 10mm into the proximal tibia, using a surgical drill equipped with a wire driver (Electric Pen Drive; DePuy Synthes J&J, West Chester, PA, United States). The wire was then removed. Either the inoculated or sterile implant was then inserted through the hole previously created via the tibial plateau into the medullary cavity. Following implant insertion, the surgical site tissues were closed in layers with 4-0 vicryl sutures (Ethicon J&J, Somerville, NJ, United States). The skin was then closed with sutures (Monosof 4-0; Covidien, Minneapolis, MN, United States). Surgical glue (Vetbond^TM^ Tissue Adhesive; 3M, Saint Paul, MN, United States) was used to close incisions that re-opened.

### Radiographic Imaging

Confirmation of the implant positioning in the medullary cavity was done using digital radiographic imaging of antero-posterior and lateral views (Ultralight 10040HF, Diagnostic X-Ray Unit; EcoRay Co. Ltd., Seoul, South Korea) and viewed with an x-ray software (FDX Console Advance, Model DR-ID 300CL; Fujifilm Holding, Tokyo, Japan). This was performed immediately postsurgery (day 1) as well as prior to necropsy on days 8 and 15.

### In-Life Observations

#### Clinical Observations and Health Assessment

Clinical signs as cage-side observations were recorded twice daily on all animals during each phase of the study. Other health assessments included clinical observations, body weight, behavioral changes, ill health, and/or mortality checks. A detailed clinical examination and body weight were performed individually at animal assignment, then weekly starting the day preceding the surgery (D-1) in addition to the day preceding necropsy. Prophylactic antibiotics were not administered given the objective of the study to produce an infection. Each animal was uniquely identified by means of a microchip.

### Terminal Procedures

#### Macroscopic Examination

Animals were euthanized at either day 8 or 15 following an overnight fasting period. The animals were anesthetized via isoflurane inhalation (3%; 3L/min O_2_; Fresenius Kabi AG, Bad Homburg, Germany) followed by exsanguination. For all animals, necropsy consisted of an external macroscopic examination, as well as a detailed internal examination with particular attention to clinical lesions and changes associated with the right hindlimb. All necropsies were performed under the supervision of a veterinary pathologist. Upon completion of the macroscopic examination, both tibiae were retained from all animals and preserved in neutral buffered formalin (10% NBF; Thermo Fisher Scientific, Waltham, MA, United States). Tibial soft surrounding tissues (muscle, fascia, and skin), knee articulations and femurs were only collected when gross findings were observed.

#### Tissue Processing

In order to prevent damage to the tissues, the implants were not removed prior to fixation, but during the trimming procedure. The tibiae, femurs and knee joints were decalcified in hydrochloric acid (Leica Biosystems, Buffalo Grove, IL, United States) overnight for a maximum of 20 h, processed by dehydration (EtOH-xylene gradients; Leica Biosystems, Buffalo Grove, IL, United States), then embedded in paraffin blocks (Leica Biosystems, Buffalo Grove, IL, United States). Using standard microtomy (Model RM2250; Leica Microsystems, Concord, ON, Canada), longitudinal histological sections were produced in the central region of the bone and prepared for microscopic examination. Sections stained with hematoxylin and eosin (H&E, Tissue-Tek Prisma, H&E Stain Kit; Sakura Finetek USA Inc., Torrance, CA, United States) and sections stained with Gram’s Twort (Poly Scientific R&D Corp., Bay Shore, NY, United States) were produced. When collected, soft tissues were processed by conventional methods, embedded in paraffin, sectioned at 4 μm and stained with H&E.

#### Histopathology Examination

All slides were examined by a board-certified anatomic pathologist under an Olympus BX43 light microscope (Olympus Corp., Center Valley, PA, United States) and photographs were captured by an Olympus DP21 camera (Olympus Corp., Center Valley, PA, United States). The histologic features of both bone and soft tissue were recorded. The specific bony changes included the presence or absence of bacteria, the type and severity of inflammation, and the presence and severity of bone necrosis and remodeling. Additionally, the specific soft tissue changes included the presence or absence of bacteria and the type and severity of inflammation.

#### Data Analysis

Numerical data obtained during the conduct of the study were subjected to calculation of group means and standard deviations. Non-numerical data obtained during the conduct of the study were reported as individual results and/or as group incidences.

## Results

### In-Life Clinical Signs and Mortality

This implantation study of *S. aureus* inoculated implants did not result into any biomaterial-related mortalities. One animal associated with high inoculation (Group 3) was euthanized on day 8 instead of 15 consequently to a procedure-related complication as a surgical incision could not be adequately re-sutured due to animal interference or self-mutilation. Nevertheless, the overall survival of animals was not affected by the insertion of unexposed sterile or inoculated implants.

### Clinical Observations

*Staphylococcus aureus* related clinical observations included slight to severe limited usage of the right hindlimb, which was observed in all animals beginning as early as day 4. Additionally, although slight swelling of the right hindlimb was observed in some control animals (Group 1), the incidence was higher in the animals from the high-dose cohort (Group 3; Table 2).

**TABLE 2:** Group incidence of *S. aureus* intramedullary implant-related macroscopic changes.

Gender	Male					
Necropsy Day	Day 8			Day 15		
Group	1	2	3	1	2	3
Dose (CFU)	0	2 × 10^6^	1 × 10^7^	0	2 × 10^6^	1 × 10^7^
Number of Animals	5	3	4	5	2	3
**Tibia^#^**	**5**	**3**	**4**	**5**	**2**	**3**
Enlargement; proximal	0	0	4	0	0	3
**Femur & Marrow^#^**	**0**	**0**	**4**	**0**	**0**	**3**
Enlargement; distal	0	0	4	0	0	3
**Knee joint^#^**	**0**	**0**	**4**	**0**	**0**	**3**
Thickening	0	0	4	0	0	3

*^#^Number examined.*

### Body Weight

**FIGURE 1:**
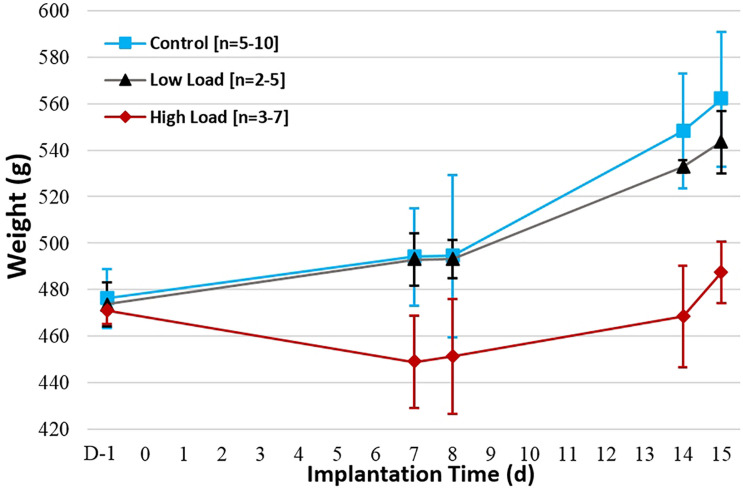
Average weight evolution per group over implantation period (days). Initial weight value is taken one day preoperatively (D-1).

All animals demonstrated changes in body weight over time following preoperative day (D-1). A decreased weight was indeed observed with high-dose animals (Group 3; ave. 487 g, day 15) as opposed to control (Group 1; ave. 562 g, day 15) and low-dose animals (Group 2; ave. 544 g, day 15), for which weight tended to increase over time (Figure 1). While the control animals and those implanted with the low-dose culture gained weight between preoperative day and day 7, all animals associated to a high inoculation lost weight over the same period. In addition, the weight increase in the case of the high-load group was also less important between days 7 and 14 compared to the other two groups.

### Digital Radiography

**FIGURE 2:**
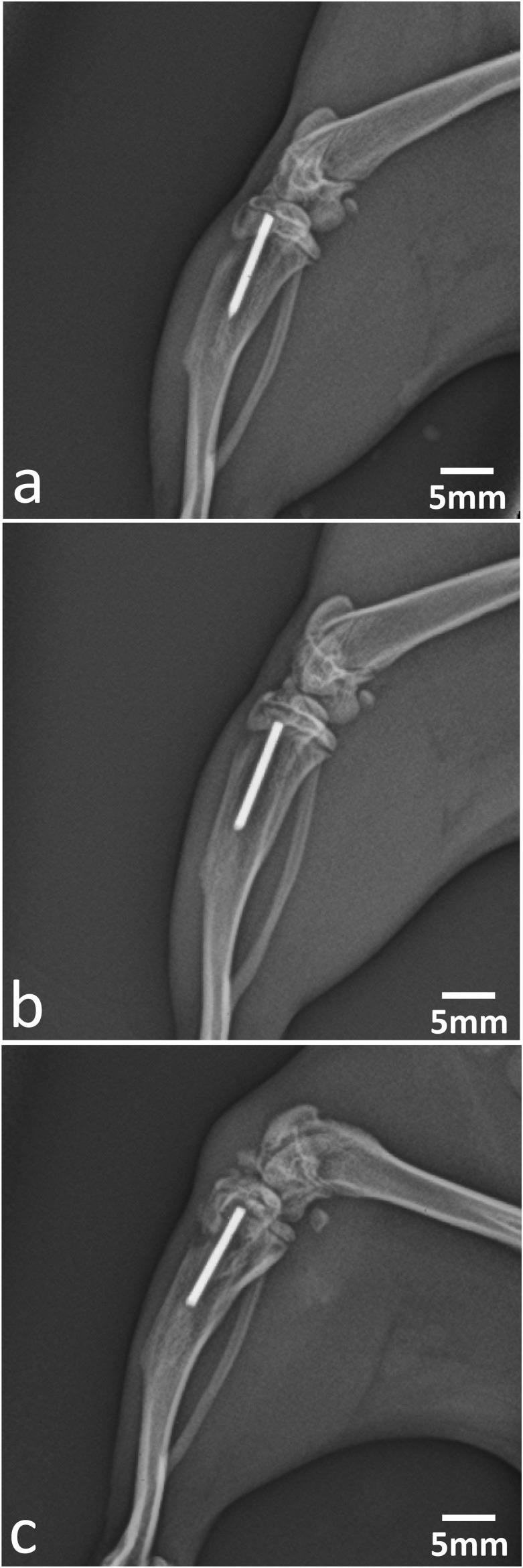
Preterminal digital radiographic imaging of intramedullary insertion sites of: **(a)** control, **(b)** low-dose, and **(c)** high-dose implants at day 15 (lateral view) showing good positioning of the K-wire implants. Similar findings were observed at day 8 (images not shown).

Digital radiographic images acquired immediately prior to necropsy confirmed the anatomical placement of the implant in the medullary cavity of the right tibia as well as the absence of migration in all cases (Figure 2). The intramedullary implants were drilled through the tibial cortex without causing any observable fracture or clinical signs immediately following surgery. No visible fractures were present prior to necropsy on days 8 and 15.

### Macroscopic Observations

Macroscopic changes, considered related to the implantation of a tibial intramedullary pin inoculated with *S. aureus*, were present in the proximal right tibia, distal right femur and right knee joint in the high-load animals. Within the right tibia, femur and knee joint, enlargement of the proximal tibia and distal femur, as well as thickening of the knee joint, were noted in 7/7 animals associated to a high inoculation load (Table 2), but were absent both in the low-dose group and in the unexposed controls.

### Microscopic Observations

#### Right Tibia and Surrounding Tissues

The implant tract was observed microscopically in the right tibia of all groups (21/22 animals; Table 3). The extent of the implant tract varied with section angle, being either partial or complete from articular surface to the boundary between metaphysis and diaphysis, including focal disruption of articular cartilage and physis from insertion. Bone remodeling changes were observed in all groups and considered related to the presence of the intramedullary pin. Minimal to moderate new bone ingrowth (22/22 animals), bone necrosis/dust (22/22 animals) and osteoblastic proliferation (7/22 animals) were noted in the medulla and located mainly along the implant tract. The location of these changes in the epiphysis, metaphysis or diaphysis varied with section angle. Osteoblastic proliferation was identified in 6/12 animals that were euthanized on day 8 and in 1/10 animals that were euthanized on day 15. This change was considered time-related. New bone ingrowth in the cortex was noted in of 6/22 animals from all groups without any dose- nor time-related incidence.

**TABLE 3:** Group incidence and severity of *S. aureus* intramedullary implant-related microscopic changes.

Gender	Male					
Necropsy Day	Day 8			Day 15		
Group	1	2	3	1	2	3
Dose (CFU)	0	2 × 10^6^	1 × 10^7^	0	2 × 10^6^	1 × 10^7^
Number of Animals	5	3	4	5	2	3
**Right Tibia^#^**	**5**	**3**	**4**	**5**	**2**	**3**
*Pin tract, present*	**5**	**3**	**4**	**5**	**1**	**3**
*Pin tract, absent*	**0**	**0**	**0**	**0**	**1**	**0**
*Bacteria*	**0**	**0**	**4**	**0**	**0**	**3**
*New bone ingrowth, medulla; epiphysis and/or metaphysis and/or diaphysis*	**5**	**3**	**4**	**5**	**2**	**3**
Minimal	0	0	0	1	0	0
Mild	3	0	1	3	1	0
Moderate	2	3	3	1	1	3
*Necrosis/dust, bone; epiphysis and/or metaphysis and/or diaphysis*	**5**	**3**	**4**	**5**	**2**	**3**
Minimal	5	3	2	2	0	2
Mild	0	0	1	3	2	1
Moderate	0	0	1	0	0	0
*Necrosis, articular cartilage*	**0**	**0**	**3**	**0**	**0**	**3**
Minimal	0	0	0	0	0	1
Mild	0	0	0	0	0	1
Moderate	0	0	2	0	0	1
Marked	0	0	1	0	0	0
*Proliferation, osteoblastic; epiphysis/metaphysis/diaphysis or pin entry*	**3**	**2**	**1**	**1**	**0**	**0**
Minimal	2	2	0	1	0	0
Mild	1	0	0	0	0	0
Moderate	0	0	1	0	0	0
*Inflammation, suppurative; epiphysis/metaphysis*	**0**	**0**	**4**	**0**	**0**	**3**
Mild	0	0	1	0	0	2
Moderate	0	0	3	0	0	1
*Inflammation, mixed; epiphysis/metaphysis*	**0**	**0**	**4**	**0**	**0**	**3**
Mild	0	0	2	0	0	1
Moderate	0	0	2	0	0	2
*Inflammation, suppurative, peri-articular*	**0**	**0**	**3**	**0**	**0**	**0**
Marked	0	0	3	0	0	0
*Inflammation, mixed, peri-articular*	**0**	**0**	**1**	**0**	**0**	**3**
Minimal	0	0	1	0	0	0
Mild	0	0	0	0	0	2
Moderate	0	0	0	0	0	1
*Granulation tissue, peri-articular*	**0**	**0**	**4**	**0**	**0**	**3**
Mild	0	0	1	0	0	3
Moderate	0	0	2	0	0	0
Marked	0	0	1	0	0	0
*New bone ingrowth, cortex; metaphysis/diaphysis*	**1**	**0**	**4**	**0**	**0**	**1**
Minimal	1	0	0	0	0	1
Mild	0	0	3	0	0	0
Moderate	0	0	1	0	0	0
**Femur & Marrow^#^**	**0**	**0**	**4**	**0**	**0**	**3**
*Inflammation; suppurative; stifle joint*	**0**	**0**	**4**	**0**	**0**	**0**
Mild	0	0	4	0	0	0
*Inflammation; mixed; stifle joint*	**0**	**0**	**0**	**0**	**0**	**3**
Mild	0	0	0	0	0	3
*Granulation tissue; stifle joint*	**0**	**0**	**4**	**0**	**0**	**3**
Mild	0	0	0	0	0	1
Moderate	0	0	1	0	0	2
Marked	0	0	3	0	0	0
*Hyperplasia, synovial cell; stifle joint*	**0**	**0**	**2**	**0**	**0**	**3**
Minimal	0	0	0	0	0	1
Mild	0	0	2	0	0	1
Moderate	0	0	0	0	0	1
*New bone ingrowth, cortex; metaphysis; diaphysis*	**0**	**0**	**1**	**0**	**0**	**1**
Mild	0	0	0	0	0	1
Moderate	0	0	1	0	0	0

*^#^Number examined.*

**FIGURE 3:**
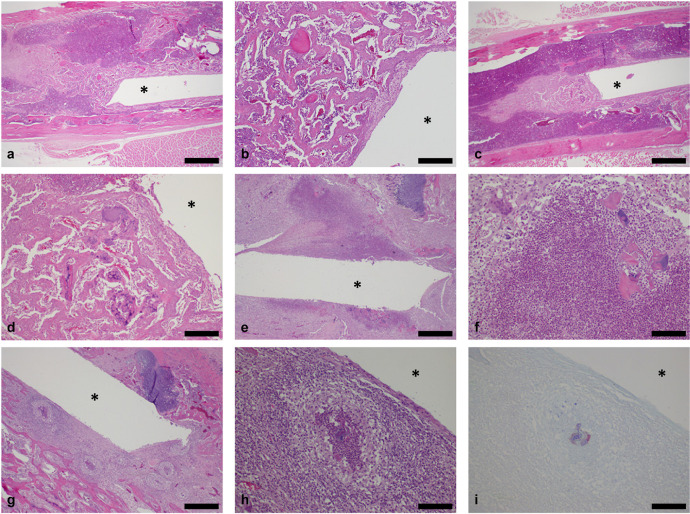
Histological longitudinal sections of right tibiae. Orientation: proximal tibia is to the right side and distal tibia is to the left side (^∗^: location of original K-wire implant tract). Control implants, day 8: Low **(a)** and high magnification **(b)** showing bone necrosis and new bone ingrowth along the pin tract at the metaphysis/diaphysis junction, extending into the diaphysis. No inflammation or bacteria were observed (H&E stain; bars: 1 mm and 200 μm respectively). Similar findings were observed at day 15 (images not shown). Low-dose implants, day 8: Low **(c)** and high magnification **(d)** showing bone necrosis/dust and new bone ingrowth along the pin tract at the metaphysis/diaphysis junction, extending into the diaphysis. No inflammation or bacteria were observed (H&E stain; bars: 1 mm and 200 μm respectively). Similar findings were observed at day 15 (images not shown). High-dose implants, day 8: Low **(e)** and high magnification **(f)** showing marked suppurative inflammation and bone necrosis along the pin tract in the metaphysis. The core of degenerated neutrophils (suppurative inflammation) is surrounded by a layer of macrophages, lymphocytes and occasional multinucleated giant cells (mixed inflammation; H&E stain; bars: 500 and 100 μm respectively). High-dose implants, day 15: Low **(g)** and high magnification **(h)** showing multifocal to coalescing micro-abcesses and bone necrosis along the pin tract in the epiphysis and metaphysis. Micro-abcesses consisting of a core of degenerated neutrophils and bacteria are surrounded by a thick layer of macrophages and mononuclear cells (H&E stain; bars: 500 and 100 μm respectively). **(i)** Intra and extracellular Gram-positive bacteria (cocci; Gram’s Twort stain; bar: 100 μm).

Microscopic changes associated with the inoculation of *S. aureus* included the presence of bacteria in the right tibia, periprosthetic and periarticular inflammation, granulation tissue and necrosis of articular cartilage, and had a dose-related incidence. The location of these changes in the epiphysis, metaphysis or diaphysis varied with section angle. Intra- and extracellular Gram-positive cocci were observed in 7/7 animals implanted with a high-inoculation load (Table 3). No bacteria were identified in the low-dose group. Periprosthetic inflammation was present in 7/7 animals from the high-inoculation load, but not in the low-dose cohort. This inflammation was characterized by multifocal to coalescing micro-abscesses consisting of a core of suppurative inflammation surrounded by a thick layer of mixed inflammation. Minimal to marked inflammation and mild to marked granulation tissue were present in the periarticular connective tissue, sometimes extending into the skeletal muscle, in 7/7 animals with high-inoculation implants, but not with the low-dose group. The type of periarticular inflammation varied in a time-dependent manner, being suppurative in animals euthanized on day 8, and mixed in animals euthanized on day 15. Minimal to marked necrosis of articular cartilage was observed in 6/7 animals with high inoculation, but not in the low-dose group. Representative images of the histological findings in the right tibia are shown in Figure 3.

#### Right Femur and Surrounding Tissues

Mild inflammation and mild to marked granulation tissue were noted in the stifle joint, including articular space, synovial membrane and periarticular connective tissue, in 7/7 animals implanted with a high inoculation load, but neither in low-dose nor in control animals. The type of inflammation was time-dependent, being suppurative in animals necropsied at day 8, and mixed in animals necropsied at day 15. These changes correlated with enlargement of the distal right femur and thickening of the right knee joint noted macroscopically. Bacteria were neither observed in the distal right femur nor the stifle joint from any group. Mild to moderate hyperplasia of synovial cells and mild to moderate cortical new bone ingrowth in the metaphysis were noted in 5/7 and 2/7 animals respectively from the high-dose group, but neither in control nor in low-dose cohorts. Various other microscopic changes were considered of low incidence, randomly distributed among groups including controls and were not considered dose-related nor procedure-related.

## Discussion

Orthopedic revision surgeries are common procedures and may be due to a number of factors, including implant migration, loosening, wear debris generation, release of metal ions and toxicity, morbidity and very importantly to surgical site infection as one of the main causes (Lerouge et al., 1996; Lentino, 2003; Puckett et al., 2010; Lichstein et al., 2014). Consequently, chronic osteomyelitis is often observed as a condition that is associated with implantation failure. Such surgical site infections are the third most frequent infections and responsible for approximately 12–16% of the nosocomial cases (Cooper, 2013), especially involving methicillin-resistant *Staphylococcus aureus* (MRSA) as the main bacterial infection type (Alt et al., 2014). In the orthopedic field, Gram-positive bacteria *S. aureus* indeed etiologically represents one of the most common causes of implant-associated osteomyelitis (Lentino, 2003). The resulting infection may lead to devitalization of hard and soft tissues, according to Shiels et al. (2016), and have an important impact on fracture malunion and non-union, implant loosening, and consequently on early device failure (Puckett et al., 2010). In order to treat the infected sites, various interventions can be undertaken and include wound debridement (Lentino, 2003), removal of implants, as well as systemic antibiotic therapy.

The development of coating therapies to better mitigate infections that arise following invasive orthopedic surgeries are of utmost importance (Alt et al., 2014). Puckett et al. (2010) have hypothesized that nanotechnology may help reducing bacterial adhesion and may represent a key to minimize orthopedic implant infection by increasing surface roughness of a biomaterial from traditional micrometer-size to nanometer size features. These parameters were indeed observed to favor osteoblast adhesion and other functions including calcium deposition and collagen secretion as opposed to fibroblast-like cells that generally prevent adequate biomaterial integration (Puckett et al., 2010). Similarly, increasing osteoblast attachment and behavior with nanostructured surfaces, while concurrently reducing the adhesion of bacteria may further be investigated: a hypothesis is that by nanotexturing a biomaterial’s topography and increasing protein adsorption, such as fibronectin, a decreased bacterial attachment can subsequently result (Puckett et al., 2010). Other postsurgical approaches may also involve antimicrobial treatment including non-rifamycin compounds such as linezolid or vancomycin, in combination with rifampin (Vergidis et al., 2011). Moreover, the developing resistance of some MRSA strains to glycopeptide antibiotics such as vancomycin have been observed (Appelbaum, 2007). Varrone et al. (2014) have hypothesized that the amidase component of autolysin may represent a molecular target of vancomycin: *S. aureus* resilience would be increased at low doses as amidase is inhibited. Investigators have evaluated various antimicrobial treatment in rat models of foreign body-associated osteomyelitis involving the tibia (Vergidis et al., 2011). Others have intended to develop passive immunization/neutralization with anti-glucosaminidase monoclonal antibodies as potential vaccines to prevent infection: mice protection against implant-associated osteomyelitis by inhibiting *S. aureus* growth seemed to be enhanced (Varrone et al., 2014).

Recently, other studies that further evaluate the dynamic interaction between MRSA and bone were also suggested with functional animal models. For example, various bone substitutes evaluated in rat models have gained importance for bone healing therapeutics and prophylaxis of infection with a slow-release of antimicrobial pharmaceuticals resulting in a combined implant with osteoconductive and bioactive properties (Shiels et al., 2016; Yang et al., 2018). New Zealand white rabbit models have also served to create infected femoral condylar and diaphyseal defects. This allowed to assess the potential for a grafted chitosan biomaterial to 3D printed polylactide-co-glycolide and HA (PLGA/HA) scaffolds implanted to reduce the progression of infection while degrading (Yang et al., 2018). In addition to these, the role of host proteins such as fibronectin adsorbed on biometals was determined in guinea pigs to explore the influence of bacterial attachment and colonization of *S. aureus* (Fischer et al., 1996). In sheep, an open fracture infection was simulated in order to evaluate the potential for silicone polymer-coated stainless steel plate constructs incorporating a cationic steroid antimicrobial to prevent infection (Williams et al., 2012). Researchers have also determined the potential to inhibit biofilm formation in a sheep unilateral transverse mid-diaphyseal tibial osteotomy repair model; this was achieved by binding a titanium compression plate to vancomycin, inoculating the plate after surgical closure and then evaluating for any sign of bone infection following fracture repair over the course of a 3-month implantation study (Stewart et al., 2012). Others have used a canine model to inject a bone cement containing gentamicin into the femoral medullary cavity to estimate if it contributed to reduce infection rates following inoculation (Petty et al., 1988).

In the present study, *Staphylococcus aureus*, one of the most common bacterial strains in implant-related infection (Arciola et al., 2012), was used to evaluate infected bone implants in rats and confirm the adequate *S. aureus* inoculation load. Bacterial presence, inflammation, morphological changes, and newly formed bone tissues were also evaluated with significant signs of infection surrounding the implants at the highest dose. Additionally, signs of osteomyelitis with intralesional bacteria were observed in the right tibia of all rats that were inoculated with 1 × 10^7^ CFUs at 8 and 15 days following surgery. Enlargement of the proximal right tibia and distal right femur, thickening of the right knee joint were noted macroscopically at necropsy in all inoculated animals with a dose-related incidence and time-related manner. These changes correlated with microscopic findings of inflammation and granulation in the stifle joint, including articular space, synovial membrane and periarticular connective tissue, sometimes extending into skeletal muscle, which were observed in the high-dose cohort. Similar changes were also described in previous rabbit studies (Odekerken et al., 2013; Alt et al., 2014; Zhou et al., 2017). Detection of osteomyelitis was consistent with previous findings observed after 2 weeks postsurgery by Odekerken et al. (2013). This type of inflammation in the stifle joint was time-dependent and dose-dependent and switched from suppurative to mixed with time, indicating chronicity. This was accompanied by decreased body weight mostly during the first week postimplant as well as swelling and limited usage of the implanted right hindlimb at the high bacterial dose. In contrast, neither the implantation of a sterile nor inoculated implant with a lower dose of inoculation resulted in the development of osteomyelitis, with absence of inflammation and clinical signs. As opposed to infected regions in inoculated implants, an appropriate healing and expected bone morphology were observed in non-contaminated rats.

## Conclusion

In this research model, the surgical implantation of a titanium wire, previously inoculated and inserted into the tibial medullary cavity of male Sprague-Dawley rats, was used to create an osteomyelitis model with inflammation in the tibia and surrounding tissues. The data indicates that a biomaterial implant in presence of a *Staphylococcus aureus* (1 × 10^7^ CFUs) inoculum was effective for the development of an osteomyelitis model of periprosthetic infection. This model may serve to investigate the safety and efficacy of biomaterial scaffolds combining local prophylactic and antimicrobial therapies such as bone substitutes, coatings, and porous implants with bioactive components against bacterial strains, in parallel to the evaluation of their structural properties for early bone fixation, healing, conduction, and osseointegration.

## Data Availability Statement

The raw data supporting the conclusions of this article will be made available by the authors, without undue reservation.

## Ethics Statement

The animal study was reviewed and approved by the Institutional Animal Care and Use Committee (IACUC), Charles River Laboratories (Laval Site), 445 Armand-Frappier Blvd., Laval, QC, Canada.

## Author Contributions

This is the result of a synergistic program conducted by our Charles River teams. SA, MA, and AD designed the study and experimental approach. YT performed the tibial surgeries and addressed surgical techniques. AD oversaw the study conduct and execution of all experimental activities as the study director. CC scored the histology sections for histopathology evaluation as the study pathologist. AD was responsible to draft the main scientific study report, while CC composed the pathology report with histology data interpretation. MA performed the literature review and drafted both the introduction and discussion. ND was responsible to review the data, text contributions, reports and manuscript to ensure scientific aspects were appropriately investigated and reported correspondingly. All authors reviewed the resulting manuscript and approved it for publication.

## Conflict of Interest

All authors were employed by Charles River Laboratories.
